# Impact of two recent extreme heat episodes on morbidity and mortality in Adelaide, South Australia: a case-series analysis

**DOI:** 10.1186/1476-069X-10-42

**Published:** 2011-05-19

**Authors:** Monika Nitschke, Graeme R Tucker, Alana L Hansen, Susan Williams, Ying Zhang, Peng Bi

**Affiliations:** 1 Department of Health, South Australia, 11 Hindmarsh Square Adelaide, South Australia 5000, Australia; 2 Discipline of Public Health, University of Adelaide, Adelaide, South Australia 5005, Australia

## Abstract

**Background:**

Extreme heatwaves occurred in Adelaide, South Australia, in the summers of 2008 and 2009. Both heatwaves were unique in terms of their duration (15 days and 13 days respectively), and the 2009 heatwave was also remarkable in its intensity with a maximum temperature reaching 45.7°C. It is of interest to compare the health impacts of these two unprecedented heatwaves with those of previous heatwaves in Adelaide.

**Methods:**

Using case-series analysis, daily morbidity and mortality rates during heatwaves (≥35°C for three or more days) occurring in 2008 and 2009 and previous heatwaves occurring between 1993 and 2008 were compared with rates during all non-heatwave days (1 October to 31 March). Incidence rate ratios (IRRs) were established for ambulance call-outs, hospital admissions, emergency department presentations and mortality. Dose response effects of heatwave duration and intensity were examined.

**Results:**

Ambulance call-outs during the extreme 2008 and 2009 events were increased by 10% and 16% respectively compared to 4.4% during previous heatwaves. Overall increases in hospital and emergency settings were marginal, except for emergency department presentations in 2008, but increases in specific health categories were observed. Renal morbidity in the elderly was increased during both heatwaves. During the 2009 heatwave, direct heat-related admissions increased up to 14-fold compared to a three-fold increase seen during the 2008 event and during previous heatwaves. In 2009, marked increases in ischaemic heart disease were seen in the 15-64 year age group. Only the 2009 heatwave was associated with considerable increases in total mortality that particularly affected the 15-64 year age group (1.37; 95% CI, 1.09, 1.71), while older age groups were unaffected. Significant dose-response relationships were observed for heatwave duration (ambulance, hospital and emergency setting) and intensity (ambulance and mortality).

**Conclusions:**

While only incremental increases in morbidity and mortality above previous findings occurred in 2008, health impacts of the 2009 heatwave stand out. These findings send a signal that the intense and long 2009 heatwave may have exceeded the capacity of the population to cope. It is important that risk factors contributing to the adverse health outcomes are investigated to further improve preventive strategies.

## Background

Excess mortality and morbidity related to heatwaves have been experienced worldwide over the past 15 years [1-4]. These have been severe enough to initiate preventive action plans in affected cities and at an international level, where efforts are supported by the World Health Organisation [5].

Adelaide, the capital city of South Australia (SA) has a population of 1,145,812 which is 73% of SA's population. It has a semi arid climate characterised in summer by hot daytime temperatures and cool nights. A recent study in Adelaide explored the potential for intensive and prolonged heat events to be associated with adverse health effects [6]. These results substantiated overseas findings that have shown increased risk of people diagnosed with renal or mental health diseases during heatwaves, as well as a substantial burden placed on ambulance services [7-9]. Increased mortality was not observed except in association with diagnosed mental health disease outcomes.

Towards the end of the warmest decade recorded in Australia, SA experienced a record breaking 15 day heatwave in March 2008 and an exceptionally long and intense heatwave in January to February 2009 [10]. Extremely strained health services were anecdotally reported on both occasions, which led to this comparison of the health impacts of these two unprecedented heatwaves. Associations between health impacts and the duration and intensity of Adelaide's heatwaves were also examined.

## Methods

### Health data

Health data from July 1993 to March 2009 were analysed. While hospital admissions and ambulance call-out data were available for the whole study period, health outcomes-specific mortality data were only available up to December 2007, and emergency department presentation data were only available from July 2003 onwards. The specific health categories used were based on the indicative results from an earlier study and from overseas findings [6,11,12].

For routinely collected hospital admissions, emergency department presentations and mortality (public and private system), the following international classifications of diseases (ICD, revisions 9 and 10) were used: total cardiovascular (ICD-9, 390-4599; ICD-10, I00-99), ischaemic (ICD-9, 410-4149; ICD-10, I20-I25), respiratory (ICD-9, 460-5199; ICD-10, J00-J99), mental (ICD-9, 290-294-9; ICD-10, F00-F999), renal (ICD-9, 580-599; ICD-10 N00-N399) and a direct heat-related category comprising dehydration, heat and sunstroke and exposure to excessive heat (ICD-9, 2765, 992, E900; ICD-10, E86, T67, X30). The pre-defined categories for ambulance call-outs were obtained from the SA Ambulance Service. Cardiac, respiratory and neurological conditions were included, while ambulance transfers between hospitals were excluded.

### Heatwave definition

An extended period of heat was categorised as a heatwave when the maximum temperature reached 35°C or above for three consecutive days or more with 35°C marking the 95^th ^percentile for maximum daily temperature for the period 1993-2009. Temperatures were obtained from the SA Bureau of Meteorology measured at a city location representative of the Adelaide metropolitan area [6]. The 2008 heatwave lasted 15 days (3 March-16 March 2008); the 2009 heatwave was defined as a 13 day episode, but included one day where the maximum temperature was just below 35°C (26 January-7 February 2009).

### Statistics

Average daily rates of adverse health effects in metropolitan Adelaide during heatwaves were compared with non-heatwave periods during the warm season (1 October to 31 March) using case series methodology [13]. Assuming that the exposure temperature is the same for the whole population, the case-series design was modified to apply at the population level using aggregated daily health outcomes in relation to acute risk and control periods. In this form, the case-series approach produces the same result as a case-crossover analysis where all non-heat wave periods in the observation period are used as control time. Case series analysis can only be used for transient exposures with a defined short term risk period. It requires that the probability of exposure is not affected by the occurrence of an outcome event. Cases are used as their own controls in non-risk periods, thus implicitly controlling for all fixed confounders [13,14]. The results are expressed as incidence rate ratios (IRR). The analysis was conducted within years; implicitly adjusting for long-term trends. Poisson regression models were fitted in Stata version 10 [15]. Each model was tested for fit, and negative binomial regression models were used to allow for over-dispersion where it occurred

The 2008 and 2009 heatwave days were included in the model as two separate dummy variables providing IRRs for the two hazard periods using the respective within warm season non-heatwave periods as control periods and adjusting for the effects of averaged heatwaves if they occurred during the same warm season. Expected cases were calculated as observed cases divided by the IRR. Excess cases were calculated by subtracting expected from observed cases.

The dose-response relationship for duration was assessed using increasing number of days within a heatwave. As heat events were sparse at the upper duration level, eight or more consecutive heatwave days were combined into one category. Health risks associated with increasing intensity were estimated for every 1°C increment in daily maximum temperature above 35°C.

## Results

**Figure 1:**
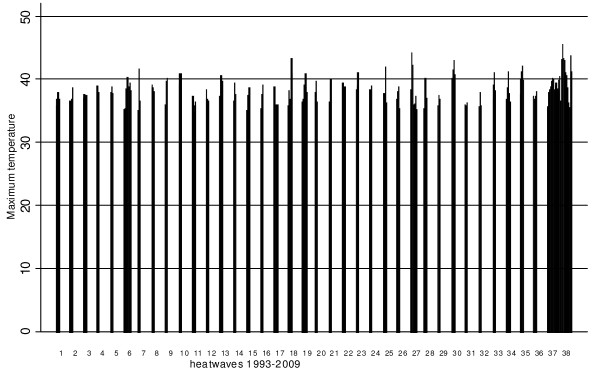
**Duration and intensity of heatwaves in Adelaide**. Distribution of daily maximum temperatures and number of days by heatwaves in Adelaide, 1993-2009.

According to the definition, a total of 38 heatwave events occurred during the study period. The distribution of maximum temperature and duration during the Adelaide heatwaves is depicted in Figure 1. When excluding the two extreme events, the mean duration of heatwaves was 3.9 days with the longest duration of eight days occurring only twice. The 2008 event was the longest heatwave on record for any Australian capital city with 15 days over 35°C and a maximum daily temperature of 40.5°C. The average minimum temperature was 22.9°C and the highest minimum was 30.2°C. In comparison, the 2009 heatwave was more intense with the maximum temperature soaring to 45.7°C. The average minimum temperature was 26.1°C and the highest minimum was 33.9°C.

Table 1 provides summary statistics for the average daily rates of ambulance call-outs, hospital admissions, emergency department presentations and mortality during heatwaves and non-heatwave periods over the past 16 years.

**Table 1:** Descriptive statistics for total and disease-specific health outcomes.

	Minimum	Maximum	Mean	SD
**Daily ambulance call-outs (5753 observation days)**	84	361	182.2	51.1

non-heat wave periods (cold seasons): 2837 days	89	331	183.9	50.6

non-heat wave periods (warm seasons): 2748 days	84	336	179.4	50.8

Heat wave period 08: 15 days:	248	303	270.9	17.6

Heat wave period 09: 13 days	243	361	291.1	36.1

Cardiac	1	66	27.1	8.0

Respiratory	0	48	16.0	6.7

Neurological	1	48	18.9	8.8

**Daily hospital separation (5753 observation days)**	202	1811	1086.5	465.2

non-heat wave periods (cold seasons): 2837 days	263	1811	1103.0	461.7

non-heat wave periods (warm seasons): 2748 days	202	1800	1066.3	469.3

Heat wave period 08: 15 days:	385	1676	1248.6	508.8

Heat wave period 09: 13 days	460	1742	1322.9	506.4

Cardio-vascular	17	137	73.0	24.6

Ischaemic heart disease	3	60	24.8	8.6

Respiratory	13	138	58.7	20.8

Mental health	5	214	35.6	13.5

Renal	1	60	21.1	9.9

Heat	0	55	1.1	1.7

**Daily Emergency Department (2101 observation days)**	653	1263	880.3	98.4

non-heat wave periods (cold seasons): 1007 days	658	1263	879.0	102.4

non-heat wave periods (warm seasons): 1020 days	653	1138	878.3	92.2

Heat wave period 08: 15 days:	889	1129	1025.4	63.3

Heat wave period 09: 13 days	905	1065	994.5	54.3

Cardio-vascular	13	63	34.8	7.7

Ischaemic heart disease	0	22	9.6	3.3

Respiratory	23	175	70.1	24.0

Mental health	0	61	22.7	16.2

Renal	5	46	19.0	5.8

Heat	0	63	2.2	3.0

**Daily mortality (5753 observation days)**	2	44	23.7	5.4

non-heat wave periods (cold seasons): 2837 days	7	43	25.3	5.4

non-heat wave periods (warm seasons): 2747 days	6	44	22.4	5.2

Heat wave period 08: 15 days:	19	31	23.2	4.2

Heat wave period 09: 13 days	15	44	28.6	9.4

Daily incidences of ambulance call-outs, hospital admissions (July 1993-March 2009), emergency department presentations (July 2003-March 2009) and mortality (total: 1993-2009; ICD specific: 1993-2006) for metropolitan Adelaide.

### Ambulance call-outs

The increases in daily total ambulance call-outs were 9.7% during the 2008 heatwave and 16% during the 2009 event compared to an average 4.4% increase during previous heatwaves (Table 2). A key difference between the two extreme events and previous averaged heatwaves was the increase in total cardiac-related call-outs. These increased by 9.8% and 12.7% respectively, while no increases in cardiac-related call-outs were observed during previous averaged heatwaves. Neurological-related call-outs increased during the two extreme events in the 65-74 year age group, but not in previous averaged heatwaves. During the 2008 heatwave, an increase in respiratory-related call-outs was also seen for the 15-64 year age group. Excess call-outs were estimated to be 360.0 (95% CI, 108.2-595.7) for the 2008 heatwave, and 517.7 (95% CI, 288.3-731.9) for the 2009 heatwave.

**Table 2:** Risk estimates for daily ambulance call-outs.

Age groups						
**Ambulance call-out categories**	**All ages**	**0-4**	**5-14**	**15-64**	**65-74**	**75+**

**Previous heatwaves**						

total ambulance	1.04 1.02-1.07	1.04 0.96-1.12	0.98 0.91-1.06	1.08 1.04-1.11	1.03 0.99-1.06	1.02 0.99-1.05

cardiac	0.98 0.95-1.02	1.12 0.80-1.57	0.64 0.26-1.58	1.02 0.95-1.08	1.02 0.95-1.10	0.94 0.89-1.00

respiratory	1.00 0.95-1.04	1.03 0.86-1.24	1.47 1.13-1.91	1.02 0.93-1.11	0.95 0.85-1.06	0.95 0.88-1.02

neurological	1.01 0.97-1.05	1.07 0.90-1.28	0.86 0.67-1.11	1.03 0.98-1.08	1.02 0.90-1.17	0.96 0.89-1.05

**Heatwave in 2008**						

total ambulance	1.10 1.03-1.17	1.09 0.87-1.35	1.13 0.92-1.37	1.12 1.03-1.21	1.13 1.03-1.24	1.04 0.97-1.13

cardiac	1.10 1.01-1.20	---	---	1.16 1.01-1.34	1.18 0.98-1.43	1.02 0.89-1.16

respiratory	1.06 0.94-1.19	1.18 0.76-1.86	0.78 0.40-1.50	1.32 1.08-1.61	0.87 0.63-1.18	0.97 0.80-1.17

neurological	1.08 0.99-1.19	1.05 0.68-1.61	0.61 0.28-1.30	1.06 0.94-1.20	1.39 1.01-1.91	1.11 0.90-1.36

**Heatwave in 2009**						

total ambulance	1.16 1.08-1.24	1.08 0.86-1.36	0.86 0.68-1.08	1.14 1.04-1.24	1.11 1.01-1.23	1.24 1.15-1.34

cardiac	1.13 1.03-1.23	---	---	1.16 0.99-1.35	1.06 0.86-1.31	1.13 0.10-1.29

respiratory	0.88 0.77-1.01	1.05 0.67-1.64	0.64 0.22-1.90	1.16 0.93-1.45	0.78 0.55-1.10	0.73 0.59-0.92

neurological	1.02 0.92-1.12	0.94 0.59-1.49	0.98 0.56-1.72	1.00 0.88-1.14	1.35 0.97-1.89	0.98 0.78-1.24

Incidence rate ratios and 95% confidence intervals (CI) for the effects of previous heatwaves, the 2008 and the 2009 heatwave, by age groups and health-specific effects. Empty cells indicate insufficient data to produce reliable estimates.

### Hospital admissions

Small (non-significant) increases in total hospital admissions were observed in all three heatwave categories for the adult age groups (Table 3). Admissions for ischaemic heart disease increased by 33% (IRR; 1.33; 95%CI, 0.99-1.80) in the 15-64 year age group during the 2009 heatwave only. While increases in mental health related admissions during previous averaged heatwaves were clearly identified across the age groups, this was not as evident during the 2008 and 2009 heatwave. Previous averaged heatwaves have shown significant increases in total renal admissions (IRR1.098; 95% CI, 1.01-1.20). This trend was repeated during the two extreme events where renal admissions were pronounced in the 5-14 and 75+ year age groups.

**Table 3:** Risk estimates for daily hospital admissions

Age groups						
**Hospital admission categories**	**All ages**	**0-4**	**5-14**	**15-64**	**65-74**	**75+**

**Previous heatwaves**						

Total	1.04 0.96-1.12	0.99 0.97-1.01	0.99 0.90-1.09	1.05 0.96-1.14	1.05 0.96-1.14	1.31 0.96-1.11

Ischaemic	0.99 0.93-1.05			1.01 0.92-1.12	1.05 0.97-1.13	0.91 0.85-0.97

Respiratory	0.95 0.88-1.03	0.88 0.79-0.98	1.00 0.89-1.13	0.99 0.89-1.11	0.94 0.88-1.01	0.89 0.83-0.95

Mental health	1.05 1.00-1.10	1.48 0.99-2.21	1.03 0.81-1.32	1.04 0.99-1.09	1.12 1.01-1.24	1.10 1.01-1.19

Renal	1.10 1.01-1.20	1.02 0.83-1.26	0.86 0.66-1.14	1.13 1.03-1.25	1.05 0.90-1.23	1.07 0.98-1.18

Direct heat	3.12 2.51-3.87	2.13 1.27-3.57	2.00 0.95-4.24	2.59 2.01-3.33	3.05 2.06-4.54	3.65 2.92-4.57

**Heatwave in 2008**						

Total	1.06 0.84-1.34	0.99 0.83-1.18	0.98 0.73-1.32	1.05 0.80-1.36	1.06 0.82-1.36	1.11 0.90-1.37

Ischaemic	1.03 0.84-1.25			0.97 0.71-1.33	1.17 0.91-1.49	0.99 0.82-1.19

Respiratory	0.98 0.77-1.24	0.83 0.59-1.2	1.02 0.71-1.47	1.06 0.77-1.45	0.91 0.72-1.13	0.96 0.80-1.15

Mental health	0.98 0.84-1.14		1.64 0.70-3.87	0.97 0.83-1.12	0.87 0.62-1.22	1.10 0.85-1.41

Renal	1.11 0.85-1.40	1.12 0.60-2.08	2.64 1.47-4.73	1.00 0.73-1.38	1.07 0.68-1.69	1.23 1.03-1.47

Direct heat	2.62 1.32-5.20			2.53 1.22-5.25	1.99 0.53-7.48	3.05 1.54-6.06

**Heatwave in 2009**						

Total	1.08 0.84-1.39	1.05 0.82-1.26	0.80 0.56-1.13	1.08 0.82-1.42	1.08 0.83-1.40	1.14 0.91-1.42

Ischaemic	1.09 0.88-1.33			1.33 0.99-1.80	0.91 0.91-1.49	0.98 0.79-1.20

Respiratory	0.95 0.73-1.22	1.03 0.74-1.42	0.65 0.41-1.04	0.10 0.71-1.41	0.82 0.64-1.05	0.98 0.84-1.14

Mental health	1.03 0.88-1.20		0.84 0.34-2.09	1.03 0.89-1.21	1.03 0.74-1.42	1.05 0.80-1.36

Renal	1.24 0.95-1.62	1.16 0.67-2.00	0.71 0.29-1.74	1.09 0.79-1.51	1.38 0.89-2.14	1.48 1.15-1.88

Direct heat	13.66 8.89-20.98			11.53 7.18-18.53	7.06 3.05-16.30	19.23 12.44-29.70

Incidence rate ratios and 95% confidence intervals (CI) of the effects of previous heatwaves, the 2008 and the 2009 heatwave by age groups and health-specific effects. Empty cells indicate insufficient data to produce reliable estimates.

A more than three-fold (IRR 3.12; 95% CI, 2.51-3.87) increase in total direct heat-related hospital admissions was observed in previous averaged heatwaves, while in 2008, the increase was less than three-fold (IRR 2.62; 95% CI, 1.32-5.20). However, during the 2009 heatwave, this increase was strengthened to nearly 14-fold (IRR 13.66; 95% CI, 8.89-20.98), with increases across the adult age range and the highest point estimate in the 75+ year age group (IRR 19.23; 95% CI, 12.44-29.7). Overall, an estimated 32.8 (95% CI, 12.8-42.8) excess heat-related hospital admissions were observed during the 2008 event and 215.0 (95% CI, 205.9-220.9) excess admissions during the 2009 event.

### Emergency Department presentations

Total emergency department presentations were significantly increased only during the 2008 heatwave (IRR 1.06; 95% CI, 1.01-1.10) (Table 4). During the 2009 heatwave, increased presentations were restricted to the 75+ year age group (IRR 1.17; 95% CI, 1.11-1.22). Previous averaged heatwave data indicate increases in renal emergency department presentations in the 0-4 year age group. During the 2008 heatwave, increases in renal presentations were seen across most age groups. An even larger impact across the age groups manifested during the 2009 heatwave.

**Table 4:** Risk estimates for daily emergency department presentations

Age groups						
**Emergency categories**	**All ages**	**0-4**	**5-14**	**15-64**	**65-74**	**75+**

**Previous heatwaves**						

Total	1.01 0.99-1.04	0.96 0.91-1.02	0.98 0.93-1.03	1.03 1.00-1.05	1.03 0.99-1.07	1.03 1.00-1.06

Ischaemic	0.91 0.82-1.01			0.90 0.76-1.06	0.99 0.81-1.20	0.88 0.75-1.03

Respiratory	0.93 0.88-1.00	0.96 0.89-1.04	1.16 0.91-1.46	0.90 0.82-0.98	0.88 0.75-1.03	0.81 0.72-0.91

Mental	1.11 1.04-1.18	1.98 0.78-4.99	0.68 0.41-1.14	1.09 1.02-1.17	1.59 1.22-2.08	1.15 0.93-1.43

Renal	1.01 0.94-1.08	1.30 1.01-1.66	0.81 0.55-1.19	0.96 0.87-1.05	1.14 0.92-1.40	1.02 0.85-1.22

Direct heat	2.68 2.19-3.28	1.58 0.86-2.91	2.29 1.21-4.32	2.99 2.24-3.99	2.60 1.74-3.89	2.73 2.20-3.37

**Heatwave in 2008**						

Total	1.06 1.01-1.10	1.02 0.92-1.13	1.04 0.95-1.14	1.07 1.02-1.12	1.09 1.02-1.15	1.03 0.97-1.08

Ischaemic	0.96 0.81-1.13			0.95 0.72-1.23	1.07 0.77-1.48	0.90 0.69-1.18

Respiratory	1.03 0.93-1.15	1.05 0.89-1.04	1.08 0.70-1.66	0.97 0.83-1.13	1.21 0.96-1.53	1.02 0.85-1.22

Mental	1.05 0.96-1.14	0.56 0.08-4.18	0.96 0.53-1.73	1.04 0.95-1.13	1.24 0.85-1.82	1.15 0.78-1.51

Renal	1.11 0.99-1.23	0.80 0.48-1.32	1.74 1.06-2.85	1.06 0.92-1.22	1.30 0.94-1.79	1.14 0.86-1.53

Direct heat	3.33 2.40-4.62	0.92 0.21-4.27	2.11 0.62-7.23	4.77 3.11-7.30	1.59 0.68-3.72	2.95 2.07-4.18

**Heatwave in 2009**						

Total	1.02 0.98-1.07	0.90 0.85-0.95	0.83 0.74-0.92	1.05 0.99-1.10	1.00 0.94-1.07	1.17 1.11-1.22

Ischaemic	1.03 0.86-1.23	---	---	1.39 1.08-1.78	0.76 0.49-1.16	0.84 0.61-1.16

Respiratory	0.77 0.68-0.88	0.72 0.62-0.83	0.64 0.36-1.14	0.86 0.73-1.02	0.80 0.61-1.05	0.83 0.68-1.00

Mental	1.04 0.95-1.13	0.62 0.83-4.60	0.96 0.58-1.59	1.05 0.96-1.10	0.66 0.40-1.09	1.18 0.85-1.64

Renal	1.39 1.26-1.54	1.51 1.02-2.23	1.25 0.71-2.21	1.32 1.16-1.50	1.21 0.87-1.71	1.68 1.29-2.10

Direct heat	12.01 9.55-15.12	3.36 1.54-7.30	3.82 1.40-10.40	12.40 8.80-17.40	9.48 6.13-14.65	15.85 12.49-20.12

Incidence rate ratios and 95% confidence intervals (CI) of the effects of previous heatwaves, the 2008 and the 2009 heatwave by age groups and health-specific effects. Empty cells indicate insufficient data to produce reliable estimates.

The most profound impact was seen on direct heat-related emergency department presentations, where the IRR for total presentations increased from 2.68 (IRR; 95% CI, 2.19-3.28) during previous averaged heatwaves to 3.33 (IRR; 95% CI, 2.40-4.62) in 2008 and to 12.01 (IRR; 95% CI, 9.55-15.12) during the 2009 event. During the 2008 heatwave, there were an estimated 77.6 (95% CI, 64.7-87.0) excess heat-related emergency department presentations. The presentations during the 2009 heatwave increased across all age groups with an overall excess of 304.4 cases (95% CI, 297.2-310.0) and the greatest excess of cases (140.5) observed in the 75+ year age group.

The 2009 heatwave was unique in that ischaemic heart disease-related emergency department presentations rose by 39% (IRR; 95% CI, 8-78%) in the 15-64 year age group. Respiratory-related emergency department presentations were generally unaffected during the 2008 heatwave, while previous averaged heatwave data and 2009 data indicate a reduction in respiratory-related emergency department presentations. As seen with hospital admissions, increases in mental health-related emergency department presentations were only observed during previous averaged heatwaves.

### Mortality

**Figure 2:**
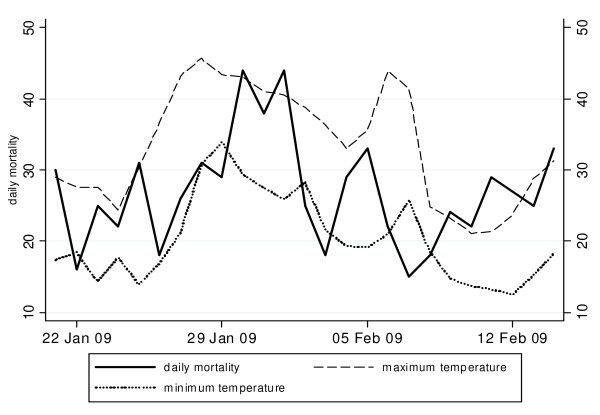
**Mortality and temperature during the 2009 heatwave**. Daily number of deaths, minimum and maximum temperatures during the 2009 heatwave (26 January-7 February 2009)

A key difference between the 2008 and 2009 heatwaves compared with previous averaged heatwaves was the effect on mortality. During previous averaged heatwaves, there was no evidence of an increase in total or age-specific mortality. Table 5 indicates that during the 2008 heatwave total mortality increased modestly, with the exception of a significant rise in the 0-4 year age group (IRR 3.23; CI, 1.30-7.99). During the 2009 heatwave, a borderline significant increase in total mortality (IRR 1.10; 95% CI, 0.99-1.22) was observed with a significant increase of 37% (IRR 1.37; 95%CI, 1.09-1.71) in the 15-64 year age group. The older age groups were unaffected. Total estimated excess mortality for the 2009 heatwave was 32.4 (95% CI,-5.5-67) with 23 (95%CI, 7.4-35.7) excess deaths in the 15-64 year age groups. Figure 2 shows the progression of mortality during the 2009 heatwave (26 January - 7 February), with a steep increase in mortality during four days when consecutive temperatures were in excess of 43°C accompanied by unusually hot nights.

**Table 5:** Risk estimates for daily mortality

Age groups						
**Total mortality**	**All ages**	**0-4**	**5-14**	**15-64**	**65-74**	**75+**

**Previous heatwaves**						

	0.98 0.94-1.22	1.15 0.81-1.63	1.18 0.62-2.28	0.98 0.90-1.07	0.99 0.91-1.09	0.97 0.92-1.01

**Heatwave in 2008**						

	1.05 0.94-1.22	3.23 1.30-7.99	3.77 0.39-36.27	1.01 0.57-2.29	1.03 0.76-1.38	1.04 0.91-1.19

**Heatwave in 2009**						

	1.10 0.99-1.22	1.53 0.54-5.31	4.33 0.45-41.66	1.37 1.09-1.71	1.15 0.86-1.52	1.00 0.88-1.15

Incidence rate ratios and 95% confidence intervals (CI) of the effects of previous heatwaves, the 2008 and the 2009 heatwaves by age groups.

### Exposure-response

Using the data from 1993-2009, a dose response relationship was evident between increasing intensity and ambulance call-outs and mortality, while hospital admissions and emergency department presentations were not associated with increasing intensity. For every 1°C increment in maximum temperature above 35°C, ambulance callouts increased by 0.7% (IRR; 95% CI, 0.4-1.1%) and mortality increased by 0.9% (IRR; 95%CI, 0.0-1.8%). A dose response was identified between duration and ambulance callouts (IRR; 1.011 95% CI, 1.007-1.016), emergency department presentations (IRR 1.009; 95% CI, 1.006-1.013), and marginally with hospital admissions (IRR 1.019; 95% CI, 1.00-1.042), but not with mortality (IRR 1.00; 95%CI, 0.99-1.01).

## Discussion

The main objective of this study was to assess the extent of health effects during the 2008 and 2009 extreme heatwaves in Adelaide and to compare to those for averaged heatwaves over the previous years. IRRs for total ambulance call-outs and for total mortality during both extreme events were higher than those for the previous averaged heatwaves, but the 2009 heatwave eclipsed the longer, but less intense 2008 event.

Although total hospital admissions and emergency department presentations were only modestly increased, specific health outcomes such as renal, mental and direct heat-related diseases were affected. Renal disease-related increases were of particular concern during the 2009 heatwave. As highlighted in a previous Adelaide study, and substantiated by international studies, heat-related dehydration appears to promote acute renal failure. Additional factors such as the physiological effects of ageing, personal behaviour, cognitive ability, pre-existing chronic disease and related medication use have also shown to contribute to acute renal disease in the elderly and the very young [8-11,16-18].

A 14-fold increase in total direct heat-related hospital admissions was recorded during the 2009 event, compared to a three-fold increase in the previous averaged heatwaves and the 2008 heatwaves. Similar findings have been reported during the concurrent heatwave in Victoria (Australia) and in other cities [17,19,20]. However, these findings could possibly be explained by greater accuracy and expertise in reporting of ICD codes for direct heat-related diseases during the 2009 heatwave than in the past because of recurring extreme heat conditions. Although, heat-related disease has the potential for poor short-term and long-term prognosis, it is largely preventable [21,22].

Surprisingly, with the exception of neurological-related ambulance call-outs in the 65-74 year age group, mental health conditions were not significantly affected during the two extreme events, contrary to what was expected based on our previous research and highlighted in the results from previous averaged heatwaves [7]. However, the lack of effects on mental health-related hospital admissions and emergency department presentations may be explained by the relatively small number of mental health-related cases during single heatwave episodes or the effect of preventive actions recently put in place to support susceptible people in Adelaide. These initiatives were introduced during the 2009 heatwave as a result of previous local investigations, and included daily telephone follow up of patients at the severe end of the mental health disease spectrum.

The 2009 event was associated with a substantial increase in hospital admissions and emergency department presentations for ischaemic heart disease, particularly in the 15-64 year age group. Cardiac-related ambulance call-outs were also significantly increased during both extreme events. This is consistent with findings from the US, where cardiovascular-related hospital and emergency department admissions were increased during heatwaves [17,23]. A recent study in Victoria found that men younger than 65 years have an increased risk of myocardial infarction when temperatures are elevated [24]. This supports our findings of an increased ischaemic heart disease risk in this younger age group. Our previous averaged heatwave data did not suggest effects on cardiac or ischaemic heart disease outcomes and this may be explained by averaging the data over relatively mild heatwaves. This absence of an effect in cardiovascular morbidity agrees with results from a European study where also no effect on heart disease admissions was found [3]. However, The sudden increase in cardiac-related mortality outside of health-care settings during the 2003 heatwave in Paris poignantly demonstrates the immediacy of cardiovascular mortality during extreme heat events suggesting that patients may die prior to presentation at hospital [25]. This is plausible considering the biological impacts of heat on blood viscosity and heart rate, especially in people with pre-existing heart conditions [26,27].

Results from previous averaged heatwaves implied that, on average total mortality during heatwaves is not higher than during non-heatwave periods. However, increased overall mortality was observed during both extreme events, with substantially greater excess deaths during the 2009 event. During the 2009 heatwave in Victoria, mortality increased by 62%, with the greatest increase in the 65+ years and over age groups which is consistent with findings in other parts of the world [19,28]. Whereas in Adelaide there was an increase in total mortality of 10%, and surprisingly, the majority of excess deaths occurred in the 15-64 year age group. When health outcome-specific mortality data becomes available, it will offer further insight into the areas of concern for this age group. Meanwhile, a possible link between the rise of ischaemic heart disease morbidity and the increase in mortality in the 15-64 year age group is only speculation. It could be argued that the absence of excess elderly deaths in Adelaide may be due to the generally high standards of care provided to the elderly. Regular telephone calls offering assistance to people at risk during the excessive heat days during the 2009 heatwave may also have contributed to the prevention of heat-related deaths in the older age groups. The higher mortality in Victoria may have been influenced by location-specific parameters. For instance, the prevalence of air-conditioning in homes is 67% in Melbourne compared to more than 80% in Adelaide [29,30]. This suggests greater preparedness in Adelaide where average summer temperatures are higher than in Melbourne (28.5°C versus 25.3°C for December, January and February). Acclimatisation to regular hot weather in Adelaide may also play a role. Similar large differences in mortality between cities have been observed overseas and the importance of location-specific parameters and investigations is strongly supported in the heatwave literature [1,31].

In accordance with overseas studies, mortality was instantly elevated on the hottest day of the 2009 heatwave (Figure 1), with only two-day latency until daily mortality peaked leaving a very small window of opportunity for preventive action [32]. This finding ties in with the observed dose response relationship between mortality and heatwave intensity but not with heatwave duration. However, the increasing duration of heatwaves was associated with increases in ambulance, hospital and emergency outcomes which may indicate that vulnerable people eventually visit health services due to accumulating heat effects.

This study design does not allow the recognition of the potential progression of heat-related disease to mortality after heatwaves have ceased, but this will be explored in a future study.

## Conclusions

The results of this study indicate increased health risks during the 2008 and 2009 heatwaves in Adelaide compared to previous averaged heatwaves. Compared to other national and international extreme heat episodes, Adelaide's health outcomes were relatively contained, but a number of important health lessons can be learned for the future. Renal and heat-related morbidity can escalate during extreme heat events and should be targeted for prevention, because of the potentially serious consequences. Unlike the findings from previous heatwave investigations, the 2009 heatwave was associated with excess deaths which are likely to be due to unprecedented intensity over consecutive days. This may indicate that the capacity to cope with heat has been exceeded during this episode. Furthermore, medical social and environmental circumstances underlying the sudden increase in ischaemic heart disease and mortality in the 15-64 year age group during the 2009 heatwave must be explored. Further studies aiming to ascertain specific risk factors that may have contributed to morbidity and mortality during recent extreme heat events in Adelaide are currently underway. Considering the likelihood of increasing incidence and severity of heat events it is crucial to interrogate local data to provide the best evidence for developing and implementing effective heat health prevention in the future.

## List of Abbreviations

SA: South Australia; ICD: international classifications of diseases; IRR: incidence rate ratio.

## Competing interests

The authors declare that they have no competing interests.

## Authors' contributions

MN has designed the study, interpreted the statistical results and wrote the first draft. GT has been involved in data acquisition and ran the statistical analysis. GT was also involved in the interpretation of data and in the revision of the manuscript. AH, SW and YZ have made substantial contribution to the first draft and to the revision. PB has contributed to the study design and edited the article. All authors read and approved the final manuscript.

## References

[B1] Vandentorren S Suzan F Medina S Pascal M Maulpoix A Cohen JC Mortality in 13 French cities during the August 2003 heat wave Am J Public Health 2004 94 1518 1520 10.2105/AJPH.94.9.1518 15333306 PMC1448485

[B2] Basu R High ambient temperature and mortality: a review of epidemiologic studies from 2001 to 2008 Environmental Health 2009 8 40 10.1186/1476-069X-8-40 19758453 PMC2759912

[B3] Michelozzi P Accetta G De Sario M D'Ippoliti D Marino C Baccini M Biggeri A Anderson HR Katsouvanni K Ballester F Bisanti L Cadum E Forsberg B Forastiere F Goodman PG Hojs A Kirchmayer U Medina S Paldy A Schindler C Sunyer J Perucci CA PHEWE Collaborative Group High Temperature and Hospitalizations for Cardiovascular and Respiratory Causes in 12 European Cities Am J Respir Crit Care Med 2009 179 383 389 10.1164/rccm.200802-217OC 19060232

[B4] Schwartz J Samet JM Patz JA Hospital admissions for heart disease. The effects of temperature and humidity Epidemiology 2004 15 755 761 10.1097/01.ede.0000134875.15919.0f 15475726

[B5] World Health Organisation Improving public health responses to extreme weather/heat-waves-EuroHEAT - Technical Summary 2009 Copenhagen: WHO Regional Office for Europe

[B6] Nitschke M Tucker Graeme Bi Peng Morbidity and mortality during heatwaves in metropolitan Adelaide MJA 2007 187 662 665 18072911 10.5694/j.1326-5377.2007.tb01466.x

[B7] Hansen A Bi P Nitschke M Ryan P Pisaniello D Tucker G The effect of heat waves on mental health in a temperate Australian city Environ Health Perspect 2008 116 1369 1375 10.1289/ehp.11339 18941580 PMC2569097

[B8] Hansen AL Bi P Ryan P Nitschke M Pisaniello D Tucker G The effect of heat waves on hospital admissions for renal disease in a temperate city of Australia Int J Epidemiol 2008 37 1359 1365 10.1093/ije/dyn165 18710886

[B9] Hansen A Bi P Nitschke M Ryan P Pisaniello D Tucker G The Effect of Heatwaves on Ambulance Callouts in Adelaide, South Australia Epidemiology 2011 22 S14 S15 [Abstracts: ISEE 22^nd ^Annual Conference, Seoul, Korea, 28 August-1 September 2010: Contributed Abstracts]

[B10] Bureau of Meteorology Annual Australian Climate Statement 2009 Melbourne 2010 http://www.bom.gov.au/announcements/media_releases/climate/change/20100105.shtml

[B11] Semenza JC McCullough JE Flanders WD McGeehin MA Lumpkin JR Excess Hospital Admissions during the July 1995 Heat wave in Chicago Am J Prev Med 1999 16 269 277 10.1016/S0749-3797(99)00025-2 10493281

[B12] Johnson H Kovats RS McGregor G Stedman J Gibbs M Walton H Cook L Black E The impact of the 2003 heat wave on mortality and hospital admissions in England Health Stat Q 2005 25 6 11 15804164

[B13] Farrington CP Whitaker HJ Semiparametric analysis of case series data J Royal Stat Soc: Series C (Appl Stat) 2006 55 553 594 10.1111/j.1467-9876.2006.00554.x

[B14] Whitaker HJ Farrington CP Spiessens B Musonda P Tutorial in Biostatistics: The self-controlled case series method Stat Med 2005 0 1 31 10.1002/sim.2302 16220518

[B15] Stata Statistical Software 2007 Version Release 10 College Station: TX: StataCorp

[B16] Kovats RS Hajat S Wilkinson P Contrasting patterns of mortality and hospital admissions during hot weather and heat waves in Greater London, UK Occup Environ Med 2004 61 893 898 10.1136/oem.2003.012047 15477282 PMC1757853

[B17] Knowlton K Rotkin-Ellman M King G Margoli HG Smith D Soloman G Trent R English P The 2006 California heat wave: impacts on hospitalizations and emergency department visits Environ Health Perspec 2009 117 61 67 10.1289/ehp.11594 PMC2627866 19165388

[B18] Rikkert MGMO Melis RJF Claassen JAHR Heat waves and dehydration in the elderly BMJ 2009 339 b2663 10.1136/bmj.b2663 19574318

[B19] Victorian Government Department of Human Services January 2009 heatwave in Victoria: an assessment of health impacts Melbourne, Victoria 2009 http://www.health.vic.gov.au/chiefhealthofficer/publications/heatwave.htm

[B20] Mastrangelo G Fedeli U Visnetin C Milan G Fadda E Spolaore P Pattern and determinants of hospitalisation during heat waves: an ecologic study BMC Public Health 2007 7 200 10.1186/1471-2458-7-200 17688689 PMC1988820

[B21] Bouchama A Knochel JP Heat stroke N Engl J Med 2002 346 1978 1988 10.1056/NEJMra011089 12075060

[B22] Argaud L Ferry T Le QH Marfisi A Ciorba D Achache P Ducluzeau R Robert D Short- and Long-term Outcomes of Heatstroke Following the 2003 Heat Wave in Lyon, France Arch Intern Med 2007 167 2177 2183 10.1001/archinte.167.20.ioi70147 17698677

[B23] Semenza JC Rubin CH Falter KH Selanikio JC Flanders D Howe HL Wilhelm JL Heat-related deaths during the July 1995 heat wave in Chicago N Engl J Med 1996 335 84 90 10.1056/NEJM199607113350203 8649494

[B24] Loughnan ME Nicholls N Tapper NJ When the heat is on: Threshold temperatures for AMI admissions to hospital in Melbourne Australia Applied Geography 2010 30 63 69 10.1016/j.apgeog.2009.08.003

[B25] Empana JP Sauval P Ducimetiere P Tafflet M Carli P Jouven X Increase in out-of-hospital cardiac arrest attended by the medical mobile intensive care units, but not myocardial infarction, during the 2003 heat wave in Paris, France Crit Care Med 2009 37 3079 84 10.1097/CCM.0b013e3181b0868f 19633540

[B26] Bhaskaran K Hajat S Haines A Effects of ambient temperature on the incidence of myocardial infarction Heart 2009 95 1760 1769 10.1136/hrt.2009.175000 19635724

[B27] Stafoggia M Forastiere F Agostini D Caranci N de'Donato F Demaria M Michelozzi P Miglio R Rognoni M Russo A Perucci CA Factors affecting in-hospital heat-related mortality: a multi-city case-crossover analysis J Epidemiol Community Health 2008 62 209 215 10.1136/jech.2007.060715 18272735

[B28] Kovats RS Hajat S Heat stress and public health: a critical review Annul Rev Public Health 2008 29 41 55 10.1146/annurev.publhealth.29.020907.090843 18031221

[B29] Australian Bureau of Statistics Household Water, Energy Use and Conservation, Victoria 2009 Canberra: ABS (ABS Catalogue No. 4602.2).

[B30] Australian Bureau of Statistics Domestic Use of Water and Energy, South Australia 2004 Canberra: ABS (ABS Catalogue No. 4618.4)

[B31] D'Ippoliti D Michelozzi P Marino C de'Donato F Menne B Katsouyanni K Kirchmayer U Analitis A Medina-Ramón M Paldy A Atkinson R Kovats S Bisanti L Schneider A Lefranc A Iñiguez C Perucci CA The impact of heat waves on mortality in 9 European cities: results from the EuroHEAT project Environ Health 2010 9 37 10.1186/1476-069X-9-37 20637065 PMC2914717

[B32] Michelozzi P de Donato F Bisanti L Ruso A Cadum E DeMaria M D'Ovidio M Costa G Perucci CA The impact of the summer 2003 heat waves on mortality in four Italian cities Euro Surveill 2005 10 pii = 556 10.2807/esm.10.07.00556-en 29208082

